# HRT, Herbal Formula, Induces G_**2**_/M Cell Cycle Arrest and Apoptosis via Suppressing Akt Signaling Pathway in Human Colon Cancer Cells

**DOI:** 10.1155/2012/871893

**Published:** 2012-07-26

**Authors:** Nam-Hui Yim, Won-Kyung Cho, Ju Hye Lee, Young Pil Jung, Hye Jin Yang, Jin Yeul Ma

**Affiliations:** KM-Based Herbal Drug Research Group, Korea Institute of Oriental Medicine (KIOM), Daejeon 305-811, Republic of Korea

## Abstract

We have demonstrated the anticancer effect of HRT in HCT116, human colon carcinoma cells. HRT inhibited cancer cell growth by causing cell cycle arrest at G_2_/M and inducing apoptosis as evidenced by DNA fragmentation assay. We found that HRT induces the activation of caspase-3, -8, and -9, whereas it reduces the level of Bcl-2 protein and results in the cleavage of PARP. Further, HRT decreased the level of phosphorylation of Akt and its downstream signals such as mTOR and GSK-3**β**. These results indicate that HRT stimulates the apoptotic signaling pathway and represses the survival and proliferation of colon cancer cells via inhibiting Akt activity. Hence, our results suggest that HRT has a potential to be developed as a therapeutic agent against colon cancer cells.

## 1. Introduction

Most colon cancer comes from high dietary fat intake and lack of adequate dietary fiber [1]. For complete eradication of colon cancer, surgical resection or chemotherapy is enforced [2], however, over 50% of patients with metastatic or locally advanced disease experience local recurrence or develop distant metastases after potential curative surgery [3]. Further, drug toxicity and resistance on chemotherapeutic agents make a struggle to treat cancer. For this reason, nontoxic dietary phytotherapy has been considered as a preventative and/or inhibitory method against cancer cells [4]. 

Hwangryunhaedok-tang (HRT; Oren-gedoku-to as Japanese name) is one of famous traditional herbal medicine being used in Asian countries, which contains four kinds of herb including *Coptis japonica*, *Scutellaria baicalensis*, *Phellodendron amurense*, and *Gardenia jasminoides*. HRT has been clinically used in Korea for the treatment of defervescence, detoxication, and inflammation for a long time [5], and a lot of studies have reported its various biological effects. They demonstrated that it has antihypertension [6], antioxidation [7], anti-inflammation [8], superior mesenteric [9], liver [6] and cardiovascular protections [10], and anticancer effects [11]. The major constituents of HRT are alkaloids including berberine, palmatine, and jatrorrhizine in *C. japonica *and *P*. *amurense*; flavonoids including baicalein and wogonin in *S. baicalensis; *iridoids including genipin and geniposide in *G. jasminoides* Ellis. These constituents contained in HRT were identified as an active ingredient of antidiabetes, antiadipogenesis, antiinflammatory, and anticancer effects [11, 12]. In this study, we investigated antiproliferation effect of HRT on various cancer cells and elucidated how HRT regulates cell cycle and apoptosis in human colon cancer cells. In normal cells, cell survival, cell cycle pathways, and cell death are well interconnected by molecular linkages that possess antagonizing functions, in contrast, deregulation of cell cycle and cellular proliferation causes unrestrained cell growth and cancer development [13]. Although some reports showed HRT has the inhibitory activities against liver cancer [11] and acute leukemia [14], it has not been reported the anticancer activity of HRT as a suppressor of survival pathway in human colon cancer. In the present study, we demonstrate that the anticancer activity of HRT comes from the synergistic effect of four constituent herbs in HRT and provide the molecular mechanism of anticancer effect induced by HRT in human colon cancer cells. 

## 2. Materials and Methods

### 2.1. Chemicals and Reagents

 Dulbecco's Modified Eagle Medium (DMEM), RPMI-1640, and Penicillin G/streptomycin were obtained from Lonza (Basel, Switzerland). Fetal bovine serum (FBS) and phosphate–buffered saline (PBS) were obtained from Hyclone (Tauranga, NZ) and WellGENE (Daegu, Republic of Korea), respectively. Ribonuclease A (RNase A), propidium iodide (PI), 3-[4, 5-dimethylthiazol-2-ly]-2, 5-diphenyltetrazolium bromide (MTT), dimethyl sulfoxide (DMSO), and ethidium bromide (EtBr) were purchased from Sigma-Aldrich (St. Louis, MO, USA). Cytotoxicity detection kit (lactate dehydrogenase, LDH) and protease and phosphatase inhibitors cocktail were purchased from Roche Diagnostics (Mannheim, Germany). Genomic DNA purification kit was purchased from Promega (Madison, USA). Primary antibodies against caspase-3, -8, -9, PARP, BID, Akt, phosphor-Akt, ERK, phosphor-ERK, phosphor-GSK3*β*, GAPDH, and secondary antibodies were purchased from Cell Signaling (Danver, MA, USA) and cyclin D1, cyclin B1, CDK7, Bcl-2, and *β*-actin were purchased from Santa Cruz Biotechnology (CA, USA). RIPA buffer and PI3K inhibitor, LY294002, were obtained from Millipore (Billerica, MA, USA) and Cell Signaling (Danver, MA, USA), respectively. Berberine-HCl and Baicalein were purchased from Korea Food & Drug Administration. Palmatine-HCl was purchased from Sigma-Aldrich (USA). Geniposide was purchased from Wako (Japan). HPLC grade solutions (water and acetonitrile) were purchased from J. T. Baker. 

### 2.2. Herb Materials and Preparation of HRT

HRT was composed of four medicinal herbs, which were listed in Table 1. The medicinal herbs were purchased from the Korea Medicine Herbs Association (Yeongcheon, Korea). HRT was described according to prescription for a 1-time dose. The mixture of medicinal herbs was extracted by heating in water of 8–10 times of herb weight for 3-4 h at 90–100°C and prepared in the form of powder by freeze-drying after concentration *in vacuo*. We obtained 3.88 g of HRT powder; 50 mg of HRT powder were dissolved in 1 mL of PBS for treatment on the cells.

### 2.3. Chromatographic Conditions

The standard compounds (Geniposide, Berberine-HCl, Palmatine-HCl and Baicalein) and powder of HRT were accurately weighed and dissolved in 60% methanol. Those were stored at 4°C and filtered through a 0.45 *μ*m membrane filter before HPLC analysis. The high performance liquid chromatography-diode array detector (HPLC-DAD) system (Hitachi Co., Japan) consisted of a pump (L-2130), autosampler (L-2200), column oven (L-2300), and diode array UV/VIS detector (L-2455). System control and data analysis were performed using EZchrom Elite software for Hitachi. The analysis of HRT and standard compounds was conducted using a Phenomenex C_18_ column (5 *μ*m, 4.6 mm × 250 mm). The mobile phase consisted of water with 0.1% Trifluoroacetic acid (TFA) (A) and acetonitrile (B) at a flow rate of 1.0 mL/min and the column temperature was maintained at 30°C. The elution conditions applied were: 0–5 min, isocratic 20% B; 5–25 min, linear gradient 20–30% B; 25–35 min, linear gradient 30–35% B; 35–45 min, linear gradient 35–40% B; 45–55 min, linear gradient 40–35% B (Table 2).

### 2.4. Cells and Culture

Various human cancer cell lines were obtained from the Korean Cell Line Bank (KCLB, Seoul, Republic of Korea) and ATCC. Cells were cultured in DMEM with 10% FBS or RPMI-1640 with 10% FBS. The media contained 100 unit/mL penicillin G and 100 *μ*g/mL streptomycin. All cells were cultured in an atmosphere of 5% CO_2_ at 37°C. 

### 2.5. MTT and LDH Assay

The cell viability assay was carried out using the MTT colorimetric assay, based on the reduction of tetrazolium salt and measurement of LDH activity in the culture supernatant. The cells were inoculated in a 96-well plate (4 × 10^3^ cells/well) and treated with extract for 24 or 48 h. After incubation, 10 *μ*L of the MTT working solution (5 mg/mL in PBS) were added to each well and incubated at 37°C for 4 h. After removing the media, formazans into the cells were dissolved with 100 *μ*L DMSO. Absorbance at 570 nm was measured using a microplate reader (Sunrise, TECAN, Männedorf, Switzerland) and cell viability was determined as the percentage of MTT reduction, assuming the absorbance of control cells as 100%. Under the same condition with MTT assay, LDH released from cells was evaluated with the commercial kit according to the manufacturer's instructions. 

### 2.6. Cell Cycle Analysis

The cells were seeded at 8 × 10^5^ cells/well in 60 mm cell culture dish, stabilized for 18 h and treated with HRT to be tested for 12, 24, and 48 h. Then cells were harvested, washed twice with cold PBS, and fixed in prechilled 70% ethanol at −20°C. The cells were resuspended in 100 *μ*L of PBS containing 10 *μ*L of RNase A (1 mg/mL) and stained with 400 *μ*L of PI (50 *μ*g/mL) for 30 min at 37°C in the dark. The DNA content of the stained cells was analyzed using CellQuest Software with the FASCalibur flow cytometry (Becton-Dickinson, Franklin Lakes, NJ, USA). 

### 2.7. Western Blot Analysis

The cells treated with HRT were washed twice with cold PBS and lysed in RIPA buffer containing protease- and phosphatase inhibitors cocktail for 30 min on ice. The lysates were centrifuged at 15,000 ×g for 20 min at 4°C and supernatant was used for western blot analysis. The same amount of protein for each sample was electrophoresed and transferred onto polyvinylidene difluoride (PVDF) membrane (Pall Corporation, USA). The membranes were blocked in tris-buffer saline containing 5% (w/v) skim milk with 0.1% Tween 20 for 1 h with a primary antibody (1 : 1000), followed by incubation with the corresponding secondary antibody (1 : 5000) at 4°C. The specific protein was detected using enhanced chemiluminescence imaging system (CoreBio, Seoul, Republic of Korea).

### 2.8. DNA Fragmentation Analysis

To investigate the apoptotic effect of HRT, we checked the appearance of oligonucleosomal DNA fragmentation by agarose gel electrophoresis. The HCT116 cells were harvested at 12, 24, and 48 h after treatment. To prepare the genomic DNA from harvested cells, we used Genomic DNA purification kit and it was performed according to the manufacturer's instructions. Genomic DNA was subjected to electrophorese on a 1.5% agarose gel impregnated with EtBr reagent for detecting ladder formation. 

### 2.9. Statistical Analysis

Data values represent means ± SD. Student's *t*-test was employed to assess the statistical significance of difference between control cells and HRT treatment cells. A *P* value of <0.05 was considered statistically significant. 

## 3. Results

### 3.1. Representative Chromatograms of Four Components in HRT

**Figure 1:**
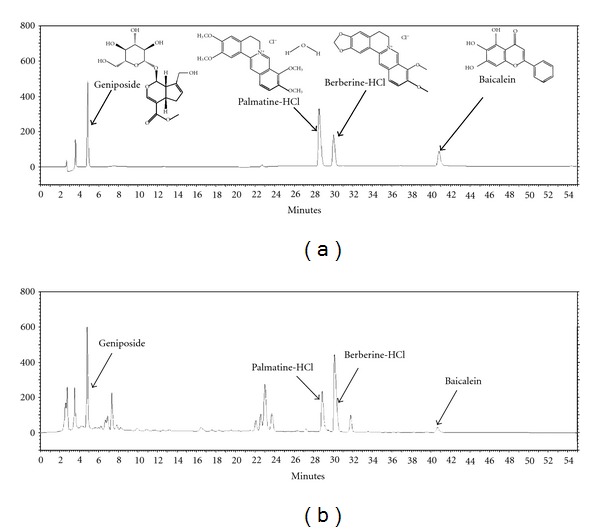
HPLC fingerprints of HRT. (a) HPLC profiling of standard components of constituent herbs contained in HRT. (b) Identification of components in HRT prescription by HPLC. HPLC chromatogram of components was monitored at 230 nm. The geniposide, palmatine-HCl, berberine-HCl, and baicalein were detected and determined as a constituent of *G. jasminoides*, *P*. *amurense*, *C. japonica*, and *S. baicalensis*, respectively. The retention times of standards for the four constituent herbs were detected at *t*_R_ 4.93, 28.54, 30.01, and 40.76 min. Four components in HRT were detected at *t*_R_ 4.88, 28.92, 30.18, and 40.71 min.

The constituents of HRT were determined by HPLC analysis and each peak of UV spectra was compared with spectra of representative standard compounds. As depicted in Figure 1(a), HPLC-DAD analysis was used to identify single representative peaks corresponding to each chemical standard of four medicinal herbs in HRT appeared at various retention times. UV spectrum analyses of reference compounds identified four constituents of HRT: berberine from *Coptis japonica *(Cj), baicalin from *Scutellaria baicalensis *(Sb), palmatine-HCl from *Phellodendron amurense *(Pa), and geniposide from *Schisandra chinensis *(Sc) (Figure 1(b)).

### 3.2. HRT Exerts Antiproliferative Effect against Human Cancer Cells

**Figure 2:**
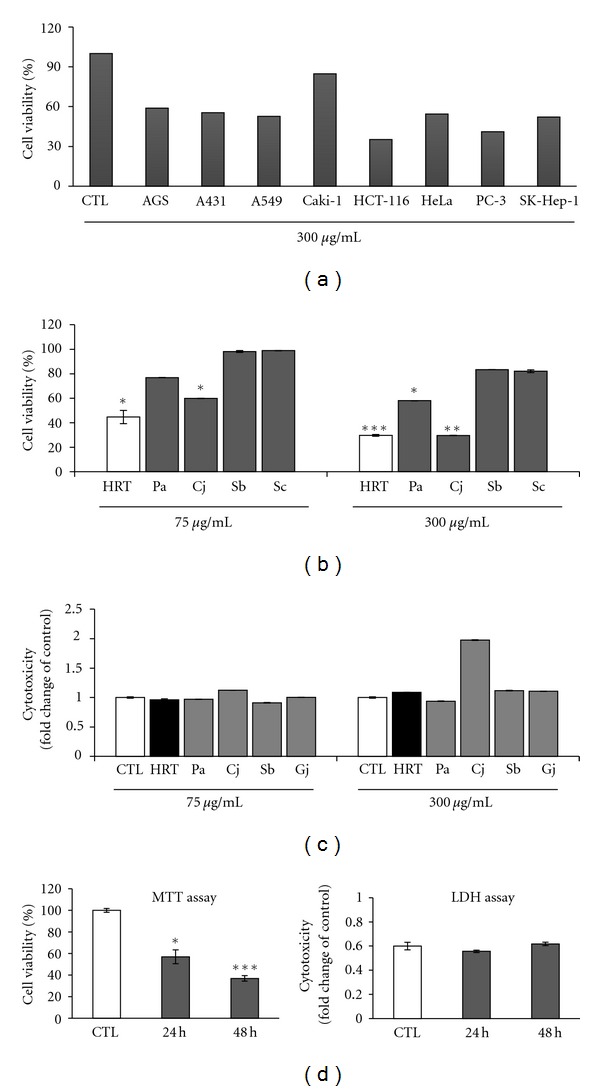
Cytotoxic effects of HRT and its constituents. (a) Stomach (AGS), epidermoid (A431), lung (A549), kidney (Caki-1), colon (HCT116), cervical (HeLa), prostate (PC-3), and liver (SK-Hep-1) cancer cells were treated with HRT (300 *μ*g/mL), incubated for 48 h and examined for antitumor effect. (b) Water extract of HRT and four constituent herbs in HRT were treated at concentration of 75 *μ*g/mL and 300 *μ*g/mL on HCT116 cells for 48 h, respectively. HRT significantly inhibited the proliferation of HCT116 cells and showed synergistic anticancer effect, compared with constituent herbs. Pa, *P. amurense*; Cj, *C*. *japonica*; Sb, *S. baicalensis*; Gj, *G. jasminoides*. (c) After treatment with HRT or four constituent herbs in HRT at concentration of 75 *μ*g/mL and 300 *μ*g/mL on HCT116 cells, the amount of LDH released into medium were measured using ELISA assay. (d) HCT116 cells were treated with HRT (300 *μ*g/mL) and incubated for 24 h and 48 h, respectively. The antiproliferative effects induced by HRT on HCT116 cells were increased with the incubation times, and results of LDH assay identified HRT induces apoptosis not necrosis effect in colon cancer cells. The data with present mean ± S.D. *P < 0.05, **P < 0.01, and ***P < 0.001 versus untreated cells.

The cytotoxicity of HRT on various cancer cells was examined using MTT assay. We tested 8 kinds of cancer cell lines, AGS (stomach), A431 (epidermoid), A549 (lung), Caki-1 (kidney), HCT116 (colon), HeLa (cervical), PC–3 (prostate), and SK-Hep-1 (liver) cells (Figure 2(a)). HRT showed the inhibitory effect on most cancer cells except for Caki-1 cells. In particular, on HCT116 cells, HRT at 300 *μ*g/mL strongly inhibited the cell growth up to 60%. On the basis of these results, the antiproliferative activity of each medicinal herb in HRT was examined in colon cancer cells at the same concentration used for HRT (Figure 2(b)). As a result, the antiproliferative effect of component herbs was weaker than that of HRT at 300 *μ*g/mL except for *C. japonica*. In particular, the extract of *S. baicalensis *or *S. chinensis *did not exhibit anticancer effect at all. *C. japonica *inhibited about 40% and 70% of the proliferation of HCT116 cells at concentrations of 75 *μ*g/mL and 300 *μ*g/mL, respectively. In contrast, HRT showed significant inhibitory effect about 56% and 71%, respectively, at same concentrations treated with *C. japonica*. When it was considered that the portion of *C. japonica *is 25% in HRT, this means HRT contains much higher anticancer activity than extract of *C. japonica* at 300 *μ*g/mL. LDH is released at late stage of apoptosis or necrosis due to cytotoxicity, for this reason, we measured LDH contents released by HRT and component herbs for detecting the cytotoxicity. As shown in Figure 2(c), extract of *C. japonica *and* S. baicalensis *showed stronger cytotoxicity than HRT at a concentration of 300 *μ*g/mL. To more define the antiproliferative activity of HRT on colon cancer cells, we checked cell viability using MTT assay and its result was compared to LDH assay on HCT116 cells. In Figure 2(d), MTT assay showed that HRT significantly inhibits cell viability up to 43% and 64% for 24 h and 48 h, respectively. In contrast, at 48 h posttreatment, LDH activity was a little increased but the ratio was insignificant compared to untreated cells (CTL). These results represent that cell death by HRT in a time-dependent manner is related with apoptotic effect. Taken together, HRT induces significant synergistic apoptosis on colon cancer cells through the complex formulation of four medicinal herbs without toxicity on normal cells.

### 3.3. HRT Causes Cell Cycle Arrest at G_2_/M Phase and Elevates Sub-G1 Population in HCT116 Cells

**Figure 3:**
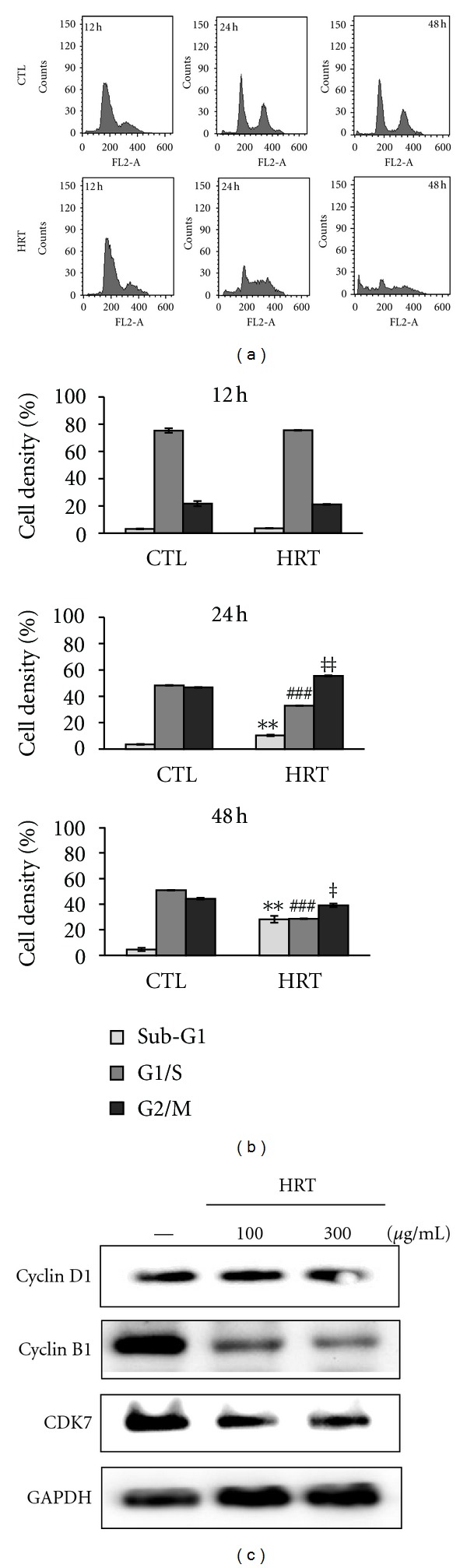
The effect of HRT on cell cycle progression in HCT116 cells. (a) The cells were treated with HRT (300 *μ*g/mL) for 12 h, 24 h, and 48 h. After fixing in 70% ethanol, the cells were stained with propidium iodide (PI) at 37°C for 30 min and analyzed by a flow cytometric system. (b) Histogram represents cell cycle analysis on HCT116 cells. ∗, #, and ‡ mean respective* P* value versus CTL. (c) Expression of cell cycle regulatory proteins in HRT-treated cells. HRT (100 or 300 *μ*g/mL) was treated in HCT116 cells for 24 h. Western blot analyses were done with anti-cyclin D1, -cyclin B1, -CDK7, and GAPDH antibodies.

To test whether HRT could affect cell cycle arrest and apoptosis of cancer cells, HCT116 cells treated with HRT (300 *μ*g/mL) for 12, 24, and 48 h were subjected to analyze flow cytometry. As shown in Figure 3(a), almost 55% of the cells treated with HRT accumulated at G_2_/M phase at 24 h posttreatment and its ratio was increased up to about 12% compared to CTL. In contrast, after the treatment with HRT for 48 h, cell population at G_2_/M phase was decreased compared to CTL, and the cells at sub-G_1_ phase were accumulated almost 28% and its ratio was increased up to about 6-times more than that of CTL. To show the total effect of HRT on HCT116 cells, the changes of DNA contents in cell cycle was calculated and presented as a line chart (Figure 3(b)). At 48 h posttreatment, the ratio of G_2_/M cells by HRT was lower than that by CTL, indicating that the portion of dead cells was increased by blocking the activation of metaphase.  Figure 3(c) shows the effect of HRT on cell cycle regulatory molecules including cyclin D1, cyclin B1, and CDK7. HRT treatment strongly decreased the expression of marker proteins associated with G_2_/M phase, such as cyclin B1 and CDK7. However, the expression of cyclin D1, a protein regulating G_1_/S phase was weakly decreased by HRT compared to GAPDH. These data support the anticancer effect by HRT is related with the increase of apoptotic cell death by G_2_/M arrest in HCT116 cells. 

### 3.4. Activation of Proapoptotic Proteins and Stimulation of DNA Fragmentation by HRT Are Attributable to Induction of Apoptotic Pathway in HCT116 Cells. 

**Figure 4:**
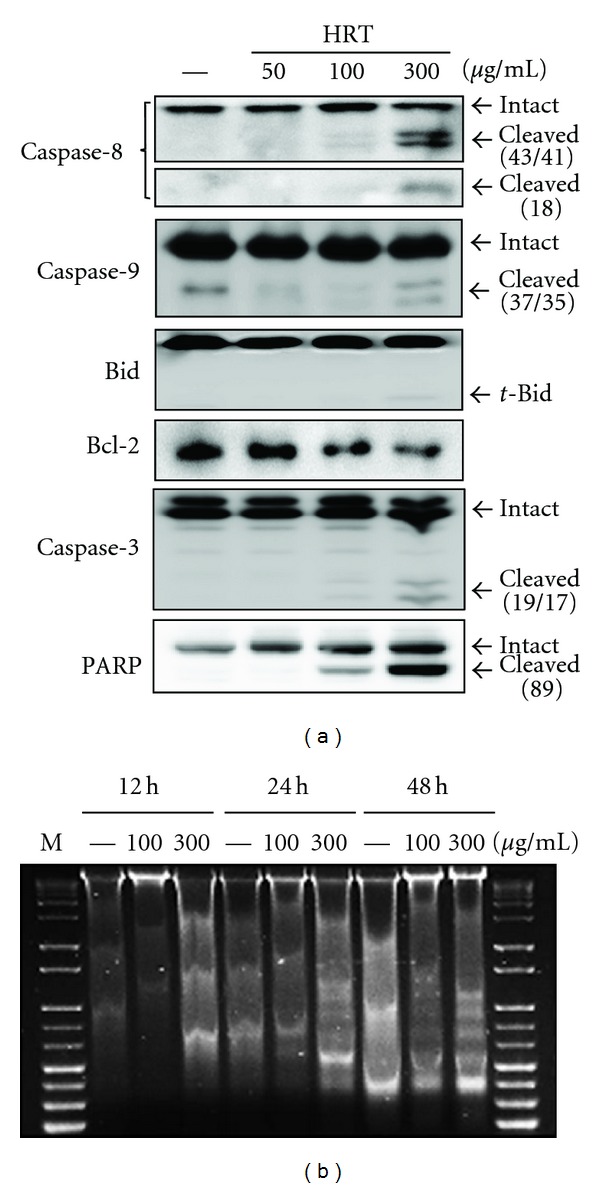
Induction of apoptosis by HRT in HCT116 cells. (a) The effect of HRT on the expression of pro-apoptotic proteins in HCT116 cells. After treatment with HRT (50, 100, or 300 *μ*g/mL) for 24 h, cell lysates were prepared for Western blot analysis against caspase-3, -8, -9, Bid, Bcl-2, PARP, and *β*-actin. (b) The DNA fragmentation was observed in HCT116 cells treated with various concentrations of HRT. DNA fragmentation with a ladder pattern is a characteristic of apoptosis.

To confirm whether cell death signals (sub-G_1_ DNA contents) induced by HRT was related to apoptosis, we examined the activation of apoptotic proteins as well as caspases using Western blot analysis. As shown in Figure 4(a), HRT stimulated caspase-3, -8, and -9 activities and cleaved forms of these caspases were clearly observed at 300 *μ*g/mL. The cleavage of PARP, a substrate of active caspase-3, was also induced on HRT treatment in a dose dependent manner. In addition, antiapoptotic factor, Bcl-2 level was decreased by HRT dose-dependently and HRT increased the level of truncated Bid (*t*-Bid), the active form of Bid, at a concentration of 300 *μ*g/mL (Figure 4(a)). In an effort to better understand the basis for apoptosis, DNA fragmentation was examined by gel electrophoresis (Figure 4(b)). The change in the amount of fragmented oligonucleosomal-length DNA was detected from 24 h after treatment of HRT in HCT116 cells. No significant ladder formation was seen until 12 h after HRT treatment but 300 *μ*g/mL of HRT initiated DNA fragmentation at 24 h. Further, 300 *μ*g/mL of HRT strongly induced DNA fragmentation at 48 h. Taken together, these results indicate that HRT stimulates apoptotic cell death through the activation of caspase-3, -8, and -9, and the interruption of DNA repair by inhibiting the action of PARP.

### 3.5. HRT Inhibits Proliferation of HCT116 Cells through Regulating Akt Signaling

**Figure 5:**
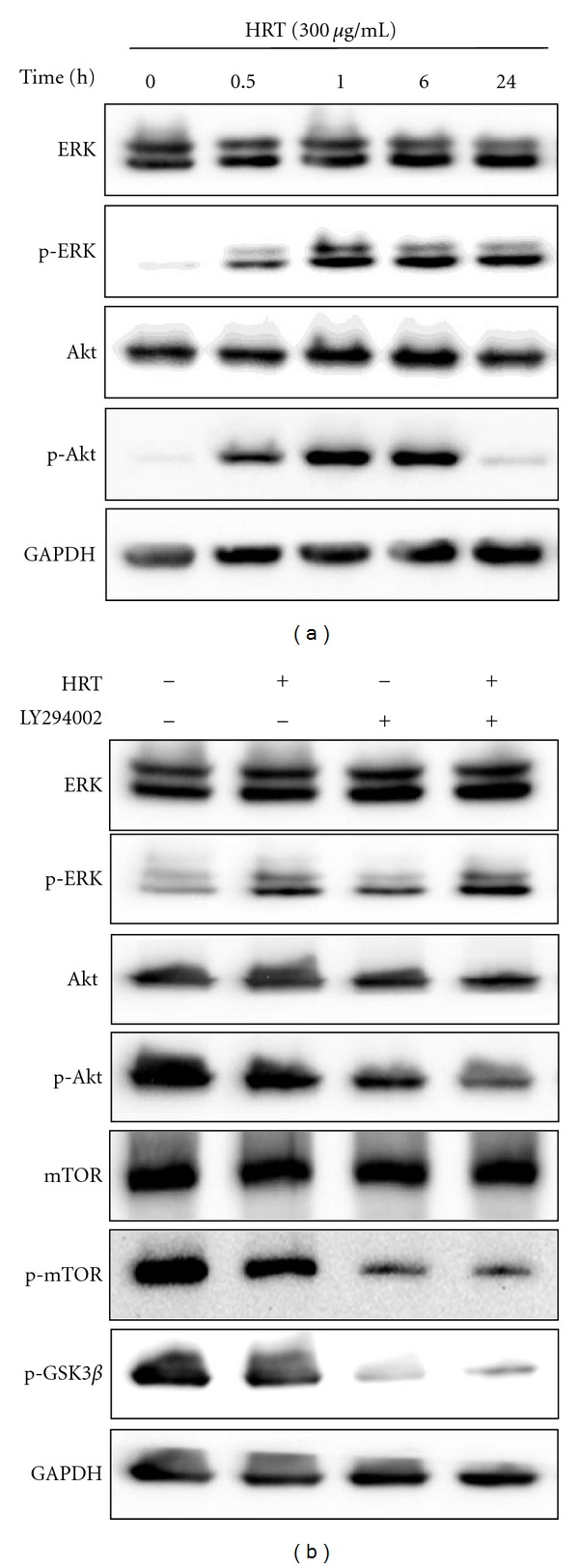
HRT inhibited cancer cell proliferation via suppressing Akt signaling. (a) The expression of phosphor-ERK or -Akt was examined in HCT116 cells treated with HRT in a time-dependent manner. HRT induced dephosphorylation of Akt not ERK at 24 h. (b) The effect of HRT on PI3K/Akt signaling pathway. Phosphorylation levels of Akt, mTOR, and GSK3*β* were examined in HCT116 cells treated with LY294002 (10 *μ*M), HRT (300 *μ*g/mL) or cotreated with LY294002 and HRT for 24 h.

In order to further demonstrate the mechanism of anticancer activity induced by HRT on HCT116 cells, we examined the factors related to cell survival. First, to investigate its effect on the activations of extracellular signal-regulated kinase (ERK) and Akt, 300 *μ*g/mL of HRT was treated after 48 h of starvation with serum free media and cells were harvested at indicated times (Figure 5(a)). As a result, the expression of phosphor-ERK (p-ERK) at 0.5 h posttreatment of HRT was detected implying ERK was activated and its activation was retained until 24 h. In contrast, Akt was shortly activated upon HRT treatment, but its activation was gradually reduced at 24 h. These results indicate HRT regulates the activation of Akt not ERK. To investigate the regulation of Akt signaling pathway by HRT in more detail, LY294002, a PI3K inhibitor, was pretreated or cotreated with HRT on HCT116 cells for 24 h and the results are shown in Figure 5(b). Both HRT and LY294002 inhibited the phosphorylation of Akt and mammalian target of rapamycin (mTOR), indicating that HRT represses the activation of Akt, thereby result in the reduced mTOR phosphorylation. Especially, the level of phosphorylation of Akt in cotreatment of HRT and LY294002 was more decreased. HRT also induced dephosphorylation of glycogen synthase kinase- (GSK-) 3*β*, implying the activation of GSK-3*β*. Taken together, HRT inhibits PI3K/Akt signaling pathway involved in the survival and proliferation of colon cancer cells.

## 4. Discussion

Although anticancer effects of traditional herbal prescription have been discovered for many years, only few studies demonstrated its inhibitory effects against cancer cell signaling pathway. HRT, which consists of four medicinal herbs, has been used for traditional oriental herbal medicine for a long time. In this study, we tested and compared the antiproliferative effect of HRT on various human cancer cells. HRT exerted strong antiproliferative effect on most cancer cells without inhibiting normal fibroblast cell proliferation. Especially, HRT exhibited more than 50% inhibitory effect on HCT116 cells. We investigated the molecular mechanism responsible for anticancer effect of HRT in HCT116 cells. Our results showed that HRT significantly restricts cancer cell survival by regulating cell cycle and inducing apoptosis. Since HRT is traditionally taken as a decoction, here, we used water extraction for further study such as preclinical study. 

Cell cycle checkpoints are important control mechanism that ensure the proper execution of cell cycle events [15]. As a checkpoint for G_2_/M phase arrest on cell cycle progression, cyclin B1 is a regulatory protein involved in mitosis and interacts with cyclin-dependent kinase 7 (CDK7) as a complex with cell division control protein kinase 2 (Cdc2) [13]. Our results showed that HRT strongly inhibits the G_2_/M phase progression and induces cell death in HCT116 cells. Consistent with that, the expressions of cyclin B1 and CDK7 were decreased by HRT in a dose-dependent manner, indicating that HRT treatment may play a critical role in G_2_/M cell cycle arrest that blocks cell proliferation and induces apoptosis. 

Apoptosis is an energy-dependent programmed cell death and its related signaling pathways have a profound effect on the progression of cancer. In cells, apoptosis can be induced by two basic parts, an intrinsic pathway activated by intracellular signals from the mitochondria and extrinsic pathway initiated by ligands engagement of cell surface death receptors [16]. The cytochrome c (cyt c) released from mitochondria activates caspase-9 and -3, and this mechanism is regulated by Bcl-2. In addition, Bid cleaved by an activated caspase-8 mediates the mitochondrial damage and the release of cyt c [17]. Active caspase-3 has been considered to be indicative of apoptosis. Another indicator of apoptosis is the proteolytic cleavage of PARP, a nuclear enzyme involved in DNA repair and stability [18]. In this study, we have shown that HRT possesses antiproliferative effect against HCT116 cells via inducing apoptosis as evidenced by activation of caspases, PARP cleavage, G_2_/M arrest and finally DNA fragmentation. Another interesting finding of the present study is that HRT blocks Akt signals not mitogen-activated protein kinase (MAPK) signals for inhibiting the proliferation of cancer cells. For getting an insight how HRT attenuated the survival of cancer cells, we examined the effect of HRT on the phosphorylation of ERK and Akt, a superfamily of MAPK and PI3K, respectively. Our results showed that phosphorylated form of Akt was increased in the early stage, however, that was sharply decreased to the basal level at 24 h posttreatment with HRT in HCT116 cells. PI3K signaling promotes tumorigenesis in cancer cells and Akt is downstream components of PI3K. Activated Akt causes tumor cell survival and inhibits of apoptosis by phosphorylating numerous downstream targets including mTOR and GSK3*β*. Especially, GSK3*β* is a primary target of Akt and inhibits anti-apoptotic molecules through inactivating GSK3*β* by phosphorylation [19]. Therefore, the inhibition of PI3K/Akt signaling by HRT can cause apoptosis in human colon cancer cells.

Some active chemical constituents were isolated from component herbs of HRT and their pharmacological effects and action mechanism were reported by previous studies [12]. Especially, in traditional Chinese medicine (TCM), the combination of *S. baicalensis *and *C. japonica* has been used clinically in the treatment of various diseases including inflammation of the eyes and gingival bleeding [20]. From these points, it is possible that the anticancer effect of HRT on human colon cancer cells may come from the synergistic action of its individual herbs or active components. 

In conclusion, this study demonstrated that a traditional herbal medicine, HRT significantly inhibits the cell-viability in several cancer cells and its antiproliferative effect is likely to be mediated by synergistic effects of individual herbal medicines. HRT effectively induces apoptosis through regulating cell cycle and activating the caspases in human colon cancer cells. In addition, at least partly, the suppression of PI3K/Akt by HRT induces dephosphorylation of mTOR and GSK3*β*, resulting in the inhibition of cancer cell proliferation. Taken together, these results suggest that HRT has a potential to be developed as a therapeutic agent against colon cancer cells after *in vivo* study using xenografts animal model. 

## Figures and Tables

**Table 1:** Composition of Hwangryunhaedok-tang (HRT) prescription.

Herbal composition	Part used	Amounts used (g)
*Coptis japonica*	Root	5
*Scutellaria baicalensis*	Root	5
*Phellodendron amurense*	Bark	5
*Gardenia jasminoides*	Fruit	5

Total amounts		20

**Table 2:** Mobile condition of chromatographic separation.

Time (min)	Solvent	
	A^a^ (%)	B^b^ (%)
0	20	80
5	20	80
25	30	70
35	35	65
45	40	60
55	30	70

^a^ Acetonitrile.

^b^0.1% trifluoroacetic acid water.
